# *The Iodine Rush*: Over- or Under-Iodination Risk in the Prophylactic Use of Iodine for Thyroid Blocking in the Event of a Nuclear Disaster

**DOI:** 10.3389/fendo.2022.901620

**Published:** 2022-05-26

**Authors:** Valeria Calcaterra, Chiara Mameli, Virginia Rossi, Giulia Massini, Mirko Gambino, Paola Baldassarre, Gianvincenzo Zuccotti

**Affiliations:** ^1^ Pediatric Department, “Vittore Buzzi” Children’s Hospital, Milan, Italy; ^2^ Pediatric and Adolescent Unit, Department of Internal Medicine, University of Pavia, Pavia, Italy; ^3^ Department of Biomedical and Clinical Sciences “L. Sacco”, University of Milan, Milan, Italy

**Keywords:** iodine, thyroid blocking, nuclear, children, prohpylaxis, hypothyroidism, hyperthyroidism, the iodine rush

## Abstract

Iodine is an essential element for the production of thyroid hormones (THs). Both deficient and excess iodine intakes may precipitate in adverse thyroidal events. Radioactive iodine (RI) is a common byproduct of nuclear fission processes. During nuclear emergencies RI may be released in a plume, or cloud, contaminating the environment. If inhaled or ingested, it may lead to internal radiation exposure and the uptake of RI mainly by the thyroid gland that absorbs stable iodine (SI) and RI in the same way. A dose of radiation delivered to the thyroid gland is a main risk factor for the thyroid cancer development. The SI prophylaxis helps prevent childhood thyroid cancer. The thyroid gland saturation with prophylactic SI ingestion, reduces the internal exposure of the thyroid by blocking the uptake of RI and inhibiting iodide organification. However, negative impact of inadequate SI intake must be considered. We provide an overview on the recommended iodine intake and the impact of SI and RI on thyroid in children and adolescents, discussing the benefits and adverse effects of the prophylactic SI for thyroid blocking during a nuclear accident. The use of SI for protection against RI may be recommended in cases of radiological or nuclear emergencies, moreover the administration of iodine for prophylactic purposes should be cautious. Benefits and risks should also be considered according to age. Adverse effects from iodine administration cannot be excluded. Precise indications are mandatory to use the iodine for thyroid blocking. Due to this natural adaption mechanism it’s possible to tolerate large doses of iodine without clinical effects, however, a prolonged assumption of the iodine when not needed can be dangerous and may precipitate in severe thyroidal and non-thyroidal negative effects.

## Introduction

Iodine is an essential component of the thyroid hormones (THs). From conception to adulthood, an inadequate iodine intake causes an impairment of THs synthesis leading to functional and developmental abnormalities of different body organs, particularly the brain (1, 2). On the other hand, an excess of iodine may precipitate in thyroid dysfunction, such as hyperthyroidism, hypothyroidism, goiter, and/or thyroid autoimmunity (3, 4).

Radioactive isotopes of iodine, such as I^131^, are included in nuclear fission products. During a nuclear disaster, radioactive iodine (RI) may be released in a plume, or cloud, contaminating the environment (5). If inhaled or ingested, it may lead to internal radiation exposure and the uptake of RI mainly by the thyroid gland that absorbs stable iodine (SI) and RI in the same way.

As reported after the Chernobyl accident in 1986, an association between exposure to RI and increased risk of thyroid cancer among children and adolescents is present (6, 7); in particular papillary and follicular thyroid carcinomas have been reported (8).

A dose of radiation delivered to the thyroid gland is a main risk factor for the thyroid cancer development (7, 9–12). The risk of radiation-associated thyroid cancer was also seen to be inversely correlated with iodine deficiency (13) and levels of SI in soil in residential areas at the time of the accident. Compared to adults, children and adolescents are at a higher risk of developing radiation-induced thyroid cancer due to different physiological and behavioral factors, such as a higher uptake rate of RI during the thyroid gland development, higher tissue dose due to the small size of the thyroid in children and different food intake (14).

During nuclear emergencies, the SI prophylaxis helps prevent childhood thyroid cancer (15, 16). The thyroid gland saturation with SI ingestion, reduces the internal exposure of the thyroid by blocking the uptake of RI and inhibiting iodide organification (16). However, adverse effects of SI intake, including iodine overload hypothyroidism, hyperthyoroidism, allergies, skin rashes, swelling of salivary glands must also be considered when a prolonged and non-appropriate assumption of the iodine occurs (17, 18).

We provide an overview on the iodine source and recommended intake and the impact of SI and RI on thyroid, focusing on children and adolescents, and discussing the benefits and adverse effects of the prophylactic SI during a nuclear accident. Defined indications for iodine prophylaxis may be useful to prevent an unjustified use of SI.

## Methods

We performed a narrative review, presenting a non-systematic summation and analysis of available literature on the topic of iodine intake and over- or under-iodination risk in prophylactic use during a nuclear accident (19). The most relevant original scientific papers, clinical trials, meta-analyses and reviews published up to February 2022, in the English language, on a specific topic, were reviewed. Case reports or series and letters were excluded. The following keywords (alone or in combination) were considered: iodine, stable iodine, radioactive iodine, thyroid, thyroid cancer, iodine thyroid blocking, iodine intake, iodine supplementation, nuclear disaster, thyroid function. The electronic databases PubMed, Scopus, EMBASE and Web of Science were used for this research. The contributions were collected by V.R., G.M., M.G., P.B. and critically analyzed with V.C. and M.C. The resulting draft was discussed by V.C. and M.C. and critically revised by G.Z. The final version was then recirculated and approved by all.

## Iodine Metabolism and Function in the Thyroid Gland

Iodine is involved in carrying out several biological functions. Although some studies have revealed, especially in recent years, the antioxidant, antimicrobial and antineoplastic properties of this element, the most important and best-known role that iodine plays in the human metabolic pathways concerns the physiology of the thyroid gland; in fact, iodine is necessary both for the synthesis of THs and for the modulation of thyroid function (20). During the first 10-12 weeks of gestation, the fetus is entirely dependent on the maternal THs. After this period, the fetal thyroid begins to be able to concentrate iodine and synthesize T3 and T4, with a mechanism that becomes more efficient starting from the 18th -20th week (2).

THs, namely 3,5,3’,5’-tetraiodo-L-thyronine (T4) and 3,5,3’-triiodo-L-thyronine (T3), whose production is mainly regulated by hypothalamic and pituitary hormones (Thyrotropin Releasing Hormone and Thyroid-Stimulating Hormone, TRH and TSH), play an essential role both for the development and for the differentiation of the cells of different organs; they are essential for neuronal development in central nervous system (CNS), in particular in the fetal period, and for somatic development.

From the biochemical point of view, T3 and T4 act as decoupling agents in the mitochondrial respiratory chain, thus increasing the cellular basal metabolism; they are also able to stimulate protein synthesis, mainly in the muscle tissue, and to regulate lipid and carbohydrate metabolism. They also increase sensitivity to catecholamines (21, 22).

Iodine is rapidly absorbed from the gastrointestinal tract, distributed in the extracellular compartment, used for the formation of THs and later eliminated mainly through the kidneys; only a small part is eliminated by sweat, saliva, tears, and bile. For this reason, the urinary iodine concentration is an excellent parameter for assessing iodine intake (23, 24).

Iodine is introduced into the cytoplasm of thyroid follicular cells thanks to the action of the NIS channel (Na-I Symporter), which introduces two sodium ions into the thyroid cell together with an Iodine molecule, *via* an active transport mechanism. In order to function properly, this transport needs the presence of a Na-K pump that maintains a higher concentration of Na ions in the extra-cellular space. Once introduced into the cytosol, iodine reaches the apical portion of the cell through several transporters a passive transport system consisting of some specific proteins, including Pendrin, regulated by the function of TSH. Here iodine undergoes the action of the enzyme thyroperoxidase (TPO), which causes oxidation and incorporation into thyroglobulin (Tg), leading to the formation of mono-iodotyrosine (MIT) and di-iodotyrosine (DIT) compounds, precursors of THs (23, 24).

The metabolism of iodine is mainly regulated by TSH, Tg and by the concentration of iodine itself: TSH is able to stimulate the production of Tg, TPO and NIS; moreover, TSH is able to modulate the intracellular vesicular traffic of TPO. All this leads to increased iodine uptake by the thyroid gland. Tg, on the other hand, acts at the genetic level, modulating the transcription of some genes, including those that code for NIS, TPO and TSH-R, thus modulating the oxidation of iodine. Uncoupled MIT or DIT residues are deiodinated by the iodotyrosine dehalogenase, that is a transmembrane protein localized at the apical pole of thyrocytes and involved in the intrathyroidal recycling of iodide (25). THs are transported outside the basolateral membrane of thyrocytes, by monocarboxylate transporter 8 (MCT8), reaching the bloodstream. The metabolism of iodine is mainly regulated by TSH, Tg and by the concentration of iodine itself: iodine deficiency stimulates TSH production, which leads to an increase in iodine uptake; excess iodine, on the other hand, inhibits the activity of TPO reducing the synthesis of THs (23, 24).

TH is essential for normal development, growth, neural differentiation, and metabolic regulation in mammals (26, 27). THs bind to thyroid hormone receptors (TRs), TRα and TRβ, which are part of the nuclear hormone receptor superfamily. These receptors also bind to enhancer elements in the promoters of target genes and can regulate both positive and negative transcription. In addition, nongenomic actions of TH that do not involve direct regulation of transcription by TR and require a plasma membrane receptor or nuclear receptors located in the cytoplasm have also been recognized (28).

## Iodine Source and Recommended Intake

### Food Sources of Iodine

The amount of iodine in foods can be variable, the causes may include seasonal effects and changes in agricultural and processing technologies that include iodine but not only, there is a great variability in the iodine content and bioavailability of soils from different regions and thus in foods. Most foods have low native iodine intake, and they don’t contribute enough to dietary intake.

The primary sources of iodine are salt, seafood, fish, algae, milk and diary. Foods from the sea, particularly certain seaweeds, are rich in iodine but their content is variable. In addition fish is not usually consumed enough to cover the daily iodine requirements. It is important to know that the iodine content in marine plants is higher than in terrestrial plants, the contents of which depend on the type of soil where they were grown (29).

Salt can be produced from underground rock salt deposits, natural brine or by evaporated seawater, the latter containing < 1mg iodine/Kg of salt (30). Salt is the preferred vehicle for iodine fortification through the use of potassium iodate or potassium iodide. The World Health Organization indicates that iodine added to salt should be estimated on the basis of the salt consumed by the population: 14 mg/Kg if salt intake is high (14g/day), 65 mg/Kg when it is low (3g/day). It is important to remember that the iodine content in the iodized salt may differ from reported content due to exposure to humidity (30).

The level of iodine in the drinking water varies according to geographical location, it reflects the amount of iodine in the soil, proximity to the sea, the water table and the agricultural runoff. The information on the content of iodine in the drinking water is often insufficient, but drinking water typically has an iodine content of 1-10 µg/L (31). In some regions the iodine content of water is too low to lead to excessive intake and potential thyroid hypertrophy, while desalinated water can cause a total loss of iodine (30).

The iodine content in plant-based food is affected by the proximity of growing area to sea water, the amount of iodine in the soil, ground, irrigation water and fertilizers containing iodine. Overall, vegetables and fruits are relatively poor sources of iodine. Seaweeds are the exception, because they have high concentration of iodine (32).

Eggs and dairy products represent a significant but variable source of iodine, milk products in fact are a major contributor of iodine but this depends on varying dairy practices. In fact their iodine content is influenced by animal feed supplements, which are used to ensure health and reproduction outcomes in dairy and beef cattle (30). In addition, the concentration of iodine in milk is closely linked to the seasons: in winter the content is higher than in summer because the milk yield is highest in the summer-autumn months (33).

Another source of iodine is represented by commercial baked goods if iodized salt is used in the technological processes by the food producer. However ionized salt is not always used in commercially processed foods (30).

Iodine in breast milk is highly variable depending on the maternal iodine intake. A well-established mechanism regulates the secretion of iodine in breast milk. In fact, in addition to the thyroid, active transport of iodide also occurs in lactating mammary glands due to a specialized form of NIS that has been identified in the healthy lactating mammary gland but not in nonlactating breasts (34). During lactation, the mammary glands concentrate iodine through an increased expression of the sodium/iodide symporter and secretes it into the breast milk. Iodide concentrated in the lactating mammary glands and secreted in milk is used by the lactating infant for the biosynthesis of thyroid hormones (34–36). High breast milk iodine concentrations have only been reported in areas with an excess of iodine intake (37). Infant formula milk must contain iodine to mimic breast milk composition. Levels of added iodine are strictly regulated: in United Europe the reference range is 15-29 µg/100 Kcal, in the United States infant formulas contain 5-75 µg/100 kcal (38, 39).

In dietary supplements, iodine is often present as potassium iodide or sodium iodide (40, 41). The iodine doses in many multivitamins/mineral supplements are usually 150 mcg, however regulation of dietary supplements is not internationally uniform and sometimes the iodine content is incorrect and it exceeds the tolerable upper intake level (42). The Dietary Supplement Label Database, created by the National Institutes of Health, reports the list of many dietary supplements that contain iodine (3, 43).

The role of goitrogens in iodine bioavailability is interesting, goitrogens are compounds with the capacity to interfere with iodine uptake and utilization by the thyroid gland and contribute to goiter disorders. Dietary goitrogens are botanical foods compounds: cassava (cyanogenic glucosides), cruciferous vegetables (glucosinolates) and soy products (flavonoids). The impact of goitrogen-source foods on thyroid status depends on the iodine content of the diet and the quantity eaten, which can be influenced by cultural practices. Nitrates, disulfides and perchlorates constitute dietary and environmental goitrogens, in fact these compounds could be a result of industrial contamination, but they may also occur naturally in soil and could therefore contaminate water and processed foods. Tobacco smoke, rich in thiocyanate, is another important goitrogenic factor (30).

Iodine intake also reflects changes in food consumption. For example, homemade foods, traditionally cooked with iodized salt, have been replaced with commercially produced foods which are not always prepared with this kind of salt, or they are too salty and consequently can have deleterious effects on the cardiovascular system. Likewise specific new dietary patterns lead to exclusion of major sources of iodine, particularly vegan, vegetarian, low salt diets, Paleo diets and lactose intolerance (44). The restriction of iodine-rich food sources such as dairy, eggs, and/or fish may increase the risk of iodine deficiency (30). Additionally, a higher consumption of soy, rich in isoflavones, can interfere with the thyroid peroxidase function (45).

### Recommended Iodine Intake

Most foods have low iodine content and diet in many regions cannot provide adequate iodine intake. Population iodine sufficiency is maintained through iodine salt fortification, indeed, in 2021, salt iodization was implemented in 124 countries and 21 have legislation permitting voluntary iodization. The consequence of this health strategy is that 88% of the global population uses iodized salt, and countries with an adequate iodine intake have doubled from 2003 to 2021. Despite this intervention on public health, 21 countries remain deficient and 13 have excessive intake (46).

The biomarkers used to monitor iodine status are: urinary iodine concentration (UIC), thyroglobulin concentration in blood and goiter rate (47). UIC is used to evaluate recent iodine intake, thyroglobulin shows thyroid activity whereas goiter rate reflects long term iodine deficiency. As reported in **Table 1**, the World Health Organization (WHO) has defined UIC categories to denote adequate iodine nutrition. However UIC measurements only provide information on the risk of developing thyroid disorders and not direct information on thyroid function (47). UIC measurements are often used to evaluate the iodine nutritional status of a population rather than individual iodine intake, it can be used to track iodine status changes over time and it is considered as a biomarker for predicting goiters among school children and is useful for facilitating interventions to address iodine excess or deficiency (48).

**Table 1:** Urinary iodine concentration in infants and children, according to World Health Organization.

Age	Urinary iodine (µg/L)	Iodine intake	Iodine nutrition
**< 2 years**	< 100 µg/L	Insufficient	Not determined
	≥ 100 µg/L	Adequate	Not determined
**School-aged children**	< 20 µg/L	Insufficient	Severe iodine deficiency
	20-49 µg/L	Insufficient	Moderate iodine deficiency
	50-99 µg/L	Insufficient	Mild iodine deficiency
	100-299 µg/L	Adequate	Optimum
	>300 µg/L	Excessive	Risk of adverse health consequences

The Recommended Dietary Allowance (RDA) for iodine intake was established by the Institute of Medicine in 2006. **Tables 2**, **3** show the reference of iodine values by age. These values include: Recommended Dietary Allowance (RDA), such as average daily level of intake sufficient to meet the nutrient requirements of nearly all healthy individuals (**Table 2**); Adequate Intake (AI), such as Intake at this level is assumed to ensure nutritional adequacy, established when evidence is insufficient to develop an RDA (**Table 2**); Tolerable Upper Intake Level (UL): Maximum daily intake unlikely to cause adverse health effects (**Table 3**) (29, 40, 41).

**Table 2:** Reference of iodine values by age, according to Institute of Medicine recommendation (29, 40, 41).

Age	Recommended Dietary Allowances	Adequate Intake
**0-6 months**	100 µg/day	110 µg/day
**7-12 months**	130 µg/day	130 µg/day
**1-5 years**	90 µg/day	Not determined
**5-12 years**	120 µg/day	Not determined
**>12 years**	150 µg/day	Not determined

**Table 3:** Recommended upper intake levels for iodine according to different institution (3, 49).

	WHO	IOM	SCF	LARN
**Premature infants**	100 µg/Kg/day	Not determined	Not determined	Not determined
**0-6 months**	150 µg/Kg/day	Not determined	Not determined	Not determined
**7-12 months**	140 µg/Kg/day	Not determined	Not determined	Not determined
**1-3 years**	50 µg/Kg/day	200 µg/day	200 µg/day	200 µg/day
**4-6 years**	Not determined	300 µg/day (4-8 Years)	250 µg/day	250 µg/day
**7-10 years**	50 µg/Kg/day (7-12 Years)	600 µg/day (9-13 Years)	300 µg/day	300 µg/day
**11-14 years**	30 µg/Kg/day (>13 Years)	Not determined	450 µg/day	450 µg/day
**15-17 years**	Not determined	900 µg/day (14-18 Years)	500 µg/day	500 µg/day

WHO, the World Health Organization; IOM, the United States Institute of Medicine; SCF, the European Union Scientific Committee on Foods; LARN, Reference intake levels for the Italian population.

The Italian Society of Human Nutrition (SINU) has carried out a Review of Reference Nutrient and Energy Intake Levels for the Italian population (LARN). This document provides food indications relating to the minimum intake of energy, micro and macronutrients, these values specifically address Italian population. **Table 4** underlines the reference of iodine values by age for the Italian population (49).

**Table 4:** Reference of iodine values by age for Italian population (49).

Age	Upper Intake Level	Adequate Intake
**6-12 months**	Not determined	70 µg/day
**1-3 years**	200 µg/day	100 µg/day
**4-6 years**	250 µg/day	100 µg/day
**7-10 years**	300 µg/day	100 µg/day
**11-14 years**	450 µg/day	130 µg/day
**15-17 years**	500 µg/day	130 µg/day

Not least were the problems related to an excess of iodine. An excess of iodine can be caused by the consumption overionized salt (>15-40 ppm) (47), drinking water, animal milk, seaweeds, dietary supplements containing iodine and/or a combination of these foods. It is for this reason that maximum iodine intakes have been defined. International reference values for upper intakes of iodine are given in **Table 1** (3). As previously stated, salt iodization, if properly implemented, is a good way to meet the needs of all population groups to prevent iodine deficiency, but sometimes the risk of localized excess iodine arises when salt fortification above the recommended level is consumed (3, 50).

## Inadequate Iodine Intake and Thyroid Disorders

Iodine plays a central role in thyroid physiology, being both a major constituent of THs and a regulator of thyroid gland function. Both iodine deficiency and excess iodine intake may cause thyroid disorders and related complications including growth retardation and neurological and intellectual impairments.

### Consequences of Iodine Deficiency

As reported in **Table 5**, iodine deficiency in pediatrics leads to a dysfunction in the synthesis of THs and can lead to various clinical conditions, according to the severity of the deficit and the age of the patient

**Table 5:** Clinical manifestations of iodine-deficiency disorder; adapted from reference (2).

**Fetus**	Abortion, Stillbirth, Increased risk of perinatal death, Cretinism
**Neonate-Infant**	Goiter, Hypothyroidism, Intellectual impairment
**Child, Adolescent**	Goiter, Hypothyroidism, Intellectual impairment, Impaired physical development

Until the 10th-12th week of gestation, when fetal thyroid tissue develops, the fetus depends to maternal THs (2). In cases in which maternal nutrition during pregnancy does not provide an adequate intake of iodine, anomalies in the fetal thyroid function may occur (51). The main consequences consist in impaired fetal neuronal development, which leads after birth to intellectual disability of varying degrees, that can be associated with other neurological and physical alterations., such as growth retardation and signs of hypothyroidism; this condition is called cretinism and can be divided into two subtypes based on the prevalent clinical manifestations (neurological *vs* myxedematous cretinism) (2).

The main manifestations of neurologic cretinism are intellectual disability, deaf mutism, gait alterations and spasticity. Hypothyroidism is not usually present. It is believed that the main cause of this type of cretinism is a state of maternal hypothyroidism present in the early stages of pregnancy followed by an adequate intake of iodine in the newborn, which therefore leads to a state of euthyroidism after birth. Myxedematous cretinism, on the other hand, manifests itself with intellectual disability, short stature, and hypothyroidism. This condition is believed to be caused by a maternal iodine deficiency in the final stages of pregnancy that is not corrected in the newborn, thus characterized by a state of hypothyroidism that continues after birth.

Severe maternal and fetal iodine deficiency has been associated with increased rates of stillbirth, abortion, congenital anomaly, and neonatal infant mortality. This effect can be reduced by up to 50 percent with iodine supplementation before conception or during pregnancy (52, 53). A study published in the Lancet in 1997 conducted in the Chinese province of Xinjiang, in an area heavily deficient in iodine and with a high infant mortality, showed that the addition of iodine in irrigation water for a period of 2-4 weeks was able to reduce the infant mortality rate from 47-106/100,000 births to 19-57/100,000 births. Similar results were obtained for neonatal mortality rate and iodine treatment with relative duration were independent predictors of infant mortality (52, 54).

A lot of studies in the literature describe how iodine deficiency is associated with varying degrees of intellectual disability. In particular, a meta-analysis conducted in this regard on several studies correlating iodine deficiency with cognitive development described how moderate-severe chronic iodine deficiency was associated with an average reduction in IQ of about 13.5 points. The consequences of mild iodine deficiencies are instead more difficult to quantify; some studies in this regard describe the presence of mild neurological developmental deficits in mildly iodine-deficient children, such as executive function, intelligence quotient (IQ) scores, reading ability, school performance, cognitive scores and language skills (1, 55–58); the correction of the intake could improve cognitive development (1). However, a recent systematic review conducted on the most recent published data on the effects of iodine supplementation in mildly-to-moderately deficient pregnant women concluded that there is neither evidence of a certain association with cognitive or neuromotor alterations, nor is there evidence that justifies iodine supplementation in pregnancy in this context (59).

During childhood, reduced intake of iodine leads to a reduced production of THs and an increase in the production of TSH by the pituitary gland. This hormone causes an increase in the size of the thyroid gland through a mechanism of thyroid cell hyperplasia, leading to a condition called goiter, which can present either as a diffuse goiter (more common in children) or as a multinodular goiter (60). Chronic TSH stimulation can lead in rare cases to gain of function mutations and therefore to hyperthyroidism conditions, more frequently in areas where there is an endemic iodine deficiency and in adult patients (61). In most cases the goiter does not reach a size that causes obstructive symptoms. In the rare cases where growth is exaggerated, signs of compression of the trachea and esophagus may occur (2).

Individuals with iodine deficiency typically have normal or reduced T3 levels, reduced T4 levels, and variably elevated TSH levels. This situation typically occurs for daily iodine intakes below 50 ug per day (2). Symptoms of iodine deficiency hypothyroidism are similar to those of hypothyroidism caused by different etiology and vary widely according to the age of the patient. In the first weeks of life, the newborn with hypothyroidism may present with lethargy, sucking disorders, bulky and bulging eyes, macroglossia, prolonged jaundice, distended abdomen, umbilical hernia, thick skin folds, significant psychomotor disability, constipation. After a few months of life, growth retardation becomes evident and a generalized picture of myxedema may rarely occur (2, 62). During childhood and adolescence myxedema, decreased growth rate, delayed bone and dental maturation, trunk-limb disproportion, lethargy, hypotonia, hyporeflexia, depressed mood, difficulties in concentration and memory, intolerance to cold, dry and pale skin, hair loss, constipation, bradycardia and changes in pubertal development may occur.

### Consequences of Iodine Excess

Iodine overdose causes less severe disability in terms of symptoms, moreover the excess intake of iodine may induce a wide spectrum of harmful consequences on thyroid function. Extrathyroidal negative effects after prolonged assumption of SI and/or acute iodine toxicity more rarely occur and are thus less discussed.

Generally, an ingestion or exposure above the limit threshold is well tolerated in healthy individuals but may cause physiological changes in vulnerable groups. Main sources of iodine in most countries exposed to high dose derived from diet (over-iodization of salt, milk, some seaweeds) but occurs also through use of iodinated contrast agents in radiologic studies and certain medications like amiodarone and povidone (iodine skin disinfectant) (3, 63).

Indeed, an acute or chronic exposure may result in hypothyroidism, hyperthyroidism, autoimmunity thyroid and goiter especially in people with preexisting thyroid disease or those previously exposed to iodine deficiency, infants and pregnant women.

When the thyroid is exposed to high dose of iodine, thyroid hormone production is temporarily suspended through synthesis of inhibitory substances acting on thyroid peroxidase activity. This condition is known as the acute Wolff-Chaikoff effect.

The proposed mechanism for the Wolff-Chaikoff effect is that iodopeptides are formed that have a temporary inhibitory effect on the synthesis of TPO mRNA and protein and, thus, on thyroglobulin iodination (64). Such an effect persists for a few days, and then intrathyroidal iodide organification is resumed and normal T4 and T3 synthesis is restored (defined as escape from the Wolff-Chaikoff effect). This is achieved by reducing the intrathyroidal concentration of inorganic iodine *via* down-regulation of NIS and thus, allows the TPO-H202 system to resume normal activity (64). However, thyroid has a natural adaption mechanism to escape from this effect recovering thyroid function in a few days by downregulation expression of the sodium-iodide symporter (NIS) thus reducing the absorption of the iodine form circulation to the thyroid and consequently the formation of iodinated inhibitory compounds.

Therefore, due to this natural adaption mechanism it’s possible to tolerate large doses of iodine without clinical effects.

As previously said people with predispositions, such as autoimmune thyroiditis, previous subtotal thyroidectomy, postpartum or subacute thyroiditis, patients treated with radioactive medication and/or iodine and some medications which interfere with iodine organification of tyrosine residues may have a defective thyroid function control mechanism resulting in an overt iodine-induced hypothyroidism (65).

Fetal and neonatal life represent a critical and susceptible period due to the immaturity of the endocrine axis and of the ability to avoid an acute Wolff-Chaikoff effect. Thus, excessive maternal iodine intake may induce goiter and hypothyroidism but the real consequences of iodine excess during pregnancy are still unclear. During infancy the principal source of iodine consumption is breast milk both exclusively breastfed infants and those being fed complementary foods (37). So, the excess iodine exposure during lactation may not only enhance the maternal susceptibility to thyroid dysfunction, but also induce subclinical and clinical hypothyroidism in their infants. During childhood however there are many controversies concerning whether a long-term iodine excess exposure may alter thyroid function and consequently the normal growth and an adequate neurological development.

Goiter is typical not only in regions characterized by iodine deficiency but also in those exposed to excessive quantities due to failure of the thyroid adaptation mechanism. Excessive iodine intake has been widely described as a risk factor for the development of thyroid autoimmunity and postpartum thyroiditis (3). The last occurs within the first year postpartum in women who were euthyroid before pregnancy, or a new occurrence of thyroid autoimmunity. Commonly, there is a hyperthyroid phase followed by a hypothyroid phase, with return to a normal thyroid function within the following year. In other cases manifestations may encompass isolated thyrotoxicosis or hypothyroxinemia (66).

Some individuals who ingest excessive amounts of iodine may develop iodine-induced hyperthyroidism, also known as the Jod-Basedow phenomenon. This is more common in patients with long-standing nodular goiters and is frequently observed following iodine supplementation in areas of a very low iodine intake. As a consequence of an increase in iodine exposure, people with thyroid nodules can elude the control of TSH and produce autonomously thyroid hormones causing hyperthyroidism. Graves’ disease is the most common cause of hyperthyroidism in iodine sufficient regions. However, several studies have evidenced that the incidence of this condition may be influenced by iodine intake but more research is required to establish whether excess iodine exposure is involved during the onset of autoimmunity of Graves’ disease (3).

Another likely consequence to consider, is that an excess iodine condition seems to increase oxidative DNA damage resulting in a major risk of developing thyroid cancer (67, 68). However, to date, it’s considered as a weak promoter and more studies are necessary to support this potential relationship.

In addition to the thyroidal effects due to excessive iodine ingestion, extrathyroidal negative effects from reiterated high level iodine intakes has also been reported, among others, on salivary glands. The salivary glands, like thyroid, are able to concentrate iodide. This glandular critical ability could cause damage, like cellular infiltration, in this extrathyroidal tissue leading to sialoadinitis (69).

Dermatological problems, such as skin rashes, acneiform eruptions and dermatitis have rarely been reported. In most cases, these adverse effects have been observed in susceptible patients, for example those with dermatitis herpetiformis or hypocomplementemic vasculitis or with preexisting thyroid disorders (69).

Acute iodine toxicity rarely occurs and clinical features from oral iodine ingestion can range from mild to severe. Mild symptoms consist of GI upset, nausea, vomiting, and diarrhea, which may progress to delirium, stupor, and shock (69).

## Radioactive Iodine and Thyroid

Following nuclear power plant (NPP) accidents, explosions expel fission products and fuel elements into the external environment, which tend to accumulate in a cloud. The most abundantly released materials are volatile elements, such as iodine isotopes, which were the ones that affected areas of Belarus, Ukraine, Russia, Poland the most after the Chernobyl NPP accident (12, 15). Indeed, iodine is one of the products of nuclear fission, which is released from NPPs operations and during nuclear weapon detonation. There are several isotopes that are released following nuclear catastrophes: I^131^ has a longer half-life, the other radioactive isotopes, present with higher core inventories, have shorter half-life, then they are important primarily in first days after NPP accident, and near the reactor. Therefore, during accidents in NPPs, one consequence is the release of radioactive iodine, compromising the health of the exposed population.

The first major example documenting the harmful action carried out by the massive release of radioiodine is the Chernobyl reactor accident of April 1986. Specifically, in the event of a nuclear accident, radioactive iodine can be released in a plume, or cloud, that contaminates the environment (air, water, soil, surfaces, plants, etc.), resulting in external exposure. Subsequent inhalation of contaminated air and ingestion of contaminated food and potable water can lead to internal radiation exposure and absorption of radioactive iodine, which primarily concerns the thyroid gland, being responsible of increased risk of thyroid alterations (70). Thus, there is no doubt that the thyroid gland is one of the most sensitive organs to, and at the highest risk of, cancer from ionizing radiation. As observed in medically irradiated patients, atomic bomb survivors, and persons involved in the Chernobyl reactor accident, radiation exposure in childhood can cause thyroid cancer and benign thyroid nodules later in life (71, 72). Autoimmune reactions involving the thyroid, thyroid atrophy, hypo- and hyperthyroidism may also be induced by radiation exposure (73).

A large amount of I^131^ accumulated in the thyroid leads to the development of hypothyroidism because of radiation-induced permanent destruction of thyroid cells. Goldsmith et al. (74) conducted regarding massive releases of I^131^ in 1945 from the Hanford plutonium production plant, which showed that, over the next 20 years, an epidemic of juvenile hypothyroidism was experienced among children living in the nearby areas. Nevertheless, it must be noted that the considered group of Hanford juveniles is not a representative sample of the general population. In fact, most of the cases of juvenile hypothyroidism found in the Hanford group were diagnosed from 1945 to 1970 (74). However, comparing the reported cases and the population under 20 years of age, it appears that juvenile hypothyroidism was associated with radioiodine exposure. In addition, this is a self-reported study and this aspect does not enable performing measurements on the general population (74).

Even in later studies (70), for example in the population affected by radioiodine emissions after the Chernobyl NPP accident, it has been suggested that for any community with large radioiodine exposure, hypothyroidism in children is a likely event, and if found, can easily be treated. Therefore, it is useful to implement targeted screening tests for such populations. Precisely for this reason, in cases where there is a smaller amount of vulnerable thyroid cells remaining, even large doses of I^131^ radiation are less likely to cause thyroid tumors. Nevertheless, it has been observed that large numbers of children exposed to a relatively low dose of radiation (less than 300 mGy) from I^131^ and perhaps other shorter-lived iodine isotopes after the 1986 Chernobyl accident developed thyroid cancer within a few years.

There has been very little research on hyperthyroidism following environmental exposure to I^131^. Some investigations in pediatric patients exposed to I^131^ from the Chernobyl fallout have focused on hypothyroidism, which is a much more common form of thyroid dysfunction, and notably two studies have reported positive results (74, 75). A recent publication based on Hiroshima and Nagasaki atomic bomb survivors who participated in a thyroid screening study (71) reported non-significant risk for developing hyperthyroidism in children exposed to external radioiodine radiation. Therefore, Hatch et al. (76) found no clear evidence of a dose-response relationship between I^131^ and the prevalence of hyperthyroidism.

Overall, exposure to ionizing radiation is a known cause responsible for the development of cancer in the human body. Especially, the accident occurred at the Chernobyl Nuclear Power Plant (NPP), which released large amounts of radioactive materials into the environment, caused an excess of thyroid cancer cases, mainly affecting children who lived in the surrounding areas of the Chernobyl NPP (77–79). Four to five years after the accident, an increase in cases of thyroid cancer was observed, particularly among younger children, aged 0-5 years at the time of exposure, while no such dramatic increase was observed in adults. Indeed, fetuses, infants, and children are at increased risk of developing radiation-induced thyroid cancer than adults because of a number of physiological and behavioral factors. On the one hand, there is a higher rate of radioiodine uptake during thyroid gland development in childhood and adolescence, and a higher tissue dose because of the small size of the thyroid gland in children (80). In addition, younger children consume a bigger amount of milk consumed than adults. In particular, after the Chernobyl accident, milk was a major source of radioiodine exposure, and its use was not immediately restricted. This was one of the leading causes for children to be disproportionately affected. The route of exposure for fetuses is placental, through the mother-child circulation; on the other hand, maternal breastfed infants are exposed to iodine that is shed in breast milk (81, 82). Several epidemiologic studies have shown evidence of a dose-dependent induction of thyroid cancers, confirming that radiation exposure is the major cause of thyroid cancer induction (83, 84). Additionally, in case of iodine deficiency, thyroid uptake of radioactive iodine is high, resulting in high doses of radiation to the thyroid. Moreover, this condition, can also increase thyroid cell proliferation rate, and consequently facilitate the occurrence of thyroid tumors (9). Hence, relationship between internal radiation exposure (beta and gamma rays) from I^131^ and the thyroid cancer risk was shown to be dose-dependent (8, 85, 86) and the risk seemed to increase linearly with the doses in the investigated range. Thus, there is strong evidence that radiation exposure is a causal factor associated with childhood thyroid cancer. Papillary and follicular carcinomas (PTCs and FTCs, respectively) in both children and adults are the most prevalent types of thyroid cancer (8). After the Chernobyl accident, almost all childhood thyroid cancers were PTCs (83, 87). Previously, a large proportion of PTCs were of solid subtype, which was a unique feature observed after the Chernobyl accident (8, 83). Subsequently, the growth pattern shifted into the classic subtype, which is less aggressive and metastatic and is quite common in sporadic childhood PTC (8, 83, 87, 88)

Thyroid cancers that resulted from exposure to ionizing radiation have provided examples revealing molecular mechanisms underlying radiation-induced carcinogenesis. Indeed, because sporadic childhood thyroid cancers found in the affected areas were quite rare, most of the cancer cases diagnosed after the Chernobyl accident could be attributable to radiation exposure. Therefore, molecular analyses were performed to understand the radiation signatures associated with malignant conversion of normal thyroid follicular cells (89).

Molecular analysis in early childhood thyroid cancer cases demonstrated a very high prevalence of genome-wide rearrangements between the RET gene and the PTC3 and PTC1 genes, all located on the same chromosome 10, producing the RET/PTC3 or RET/PTC1 rearrangements (90). RET/PTC rearrangements, produced by paracentric (intrachromosomal) inversion within the long arm of chromosome 10, are now recognized as predominant driver mutations in childhood papillary thyroid cancers, both radiation-related and sporadic (91–93). This process gives rise to the fusion genes between the thyrosine kinase domain of the RET gene and the amino terminal region of the PTC gene. The product of these gene rearrangements, namely RET/PTC fusion proteins, are persistently active and stimulate the mitogen-activated protein kinase (MAPK) pathway and other signaling cascades in a ligand-independent manner (93–96). It was thought that this type of gene rearrangement (RET/PTC) represented the signature of ionizing radiation, since other types of mutations related to the development of PTC, such as the one in the BRAF gene, occurred especially in adult cases, and its prevalence in PTCs cases in childhood after the Chernobyl NPP accident was below 10% on average (8, 97). Albeit literature data reported that RET/PTC rearrangements were induced by radiation exposure, *in vitro* studies are unable to assess what the spontaneous incidence of RET/PTC rearrangements really is, because their frequency, in the absence of genotoxic stimuli, is too low. Numerous independent groups have assessed the prevalence of RET/PTC rearrangements in childhood thyroid cancers after the Chernobyl accident, but only a few studies have made comparisons with the frequency of RET/PTC rearrangements in sporadic childhood PTCs (98–100). The data collected seem to show that the frequency of rearrangements, particularly that of the RET/PTC1 rearrangement, was comparable between the two groups of patients with radiation-induced and sporadic tumors (97, 101–103). This suggests that RET/PTC rearrangements in radiation-related cases might not be the signature of radiation, but rather, radiation exposure might unveil RET/PTC rearrangements that occurred spontaneously (89). In fact, considering that thyroid cancers in children started to occur 4-5 years after the Chernobyl accident, it would be reasonable to assume that thyroid follicular cells with RET/PTC rearrangements already existed, and radiation exposure could provide a chance for cells with such tumor signatures to proliferate (89).

**Figure 1:**
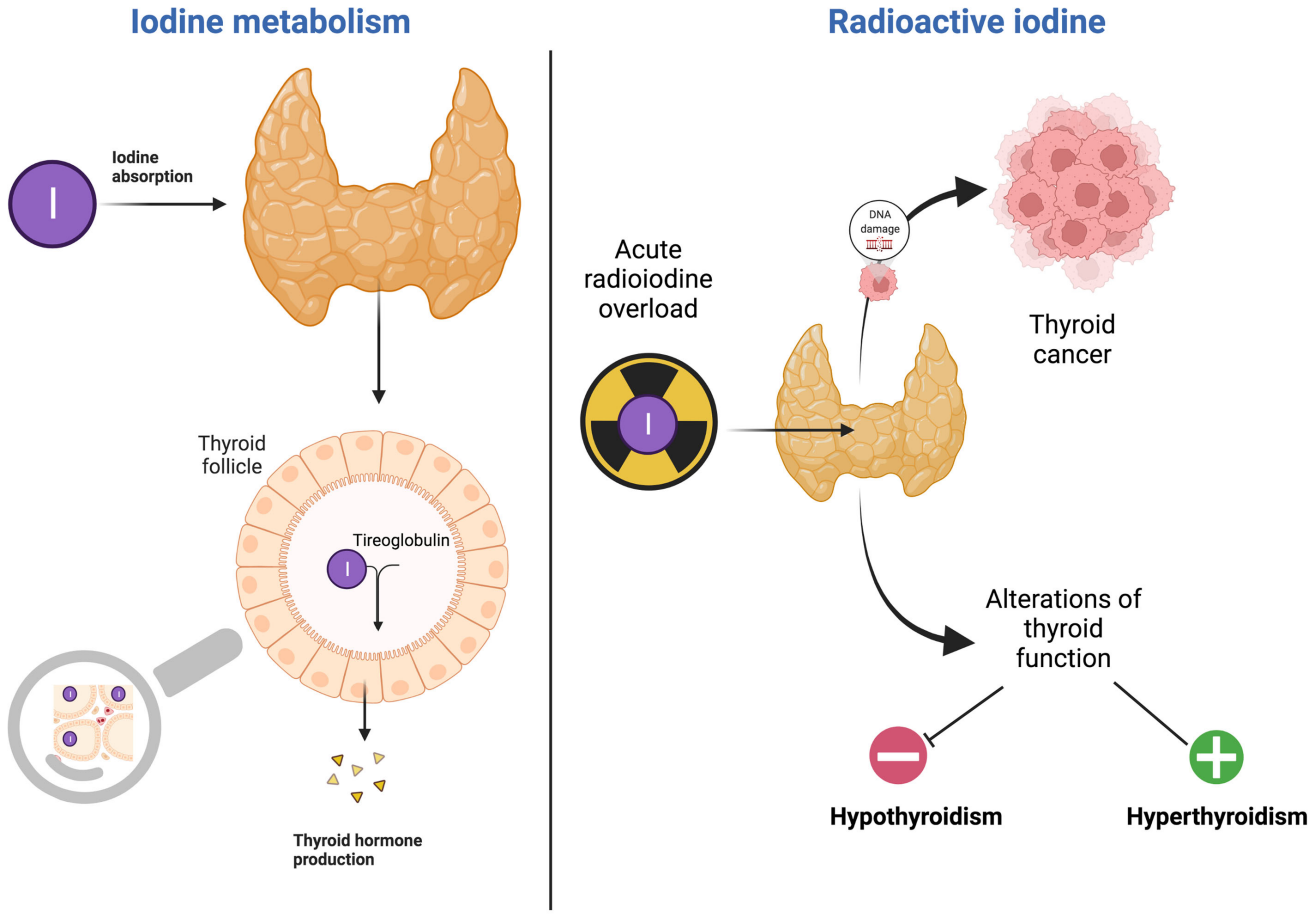
Radioiodine impact on the thyroid disorders (created by using Biorender).

In **Figure 1**, RI effects on thyroid damage are schematized.

## Use of Iodine for Thyroid Blocking in the Nuclear Disaster: Benefits and Risks

Because the thyroid uses iodine to produce THs, iodine is an organic compound that is found in high concentrations in the thyroid gland, which, however, cannot discern between radioactive and stable iodine (5). Hence, following a nuclear accident and the release of large amounts of radioactive iodine, if this is inhaled or ingested, the thyroid will absorb it in just the same way as stable iodine.

**Figure 2:**
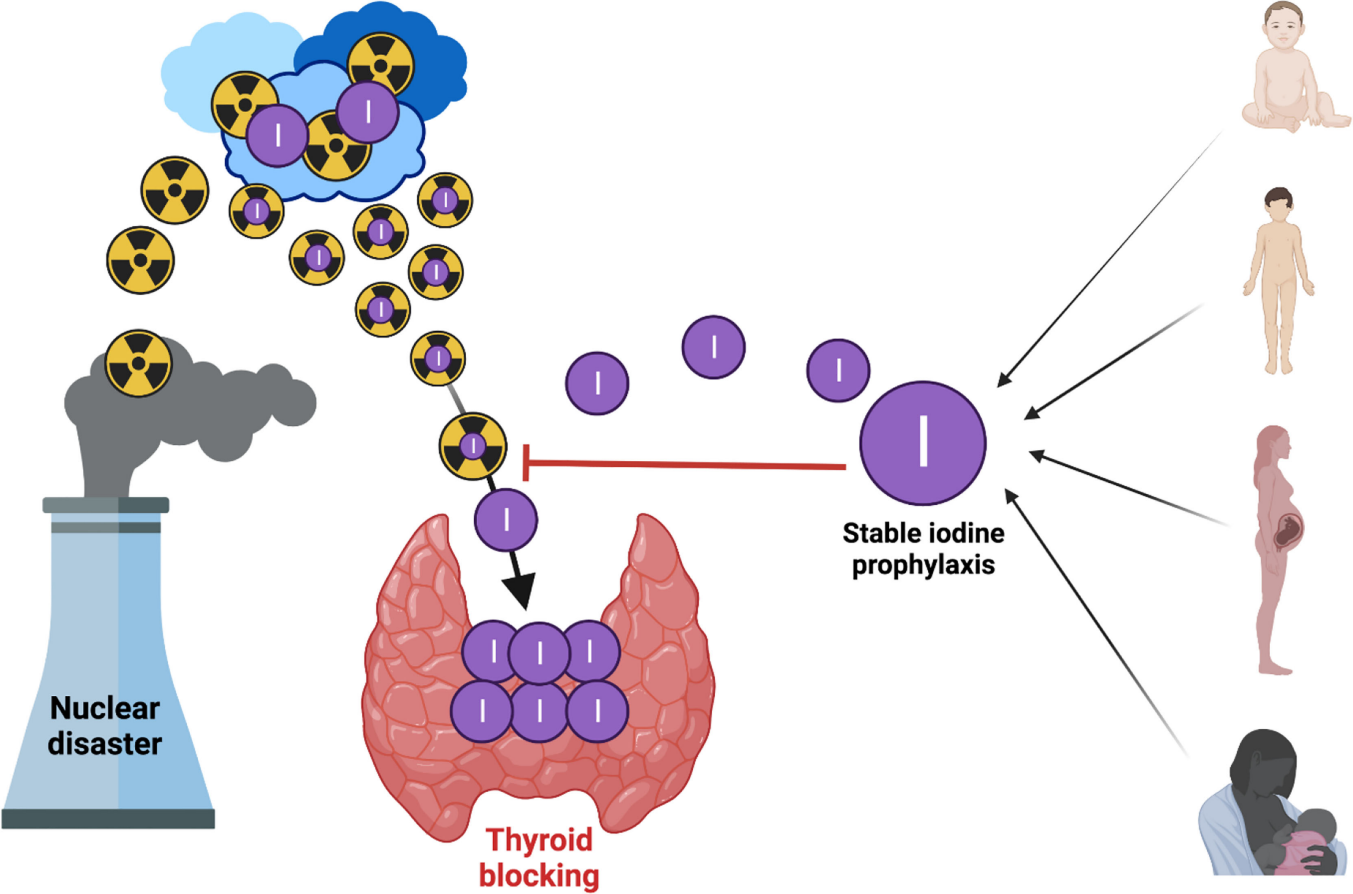
Blocking thyroid with oral iodine to prevent uptake of radioactive iodine isotopes (created by using Biorender).

Blocking thyroid by oral administration of potassium iodide (KI) is considered a practical and successful protective measure for the general population in an emergency (11, 15, 104, 105). Stable oral iodine saturates (‘blocks’) the thyroid and prevents the uptake of radioactive iodine isotopes. Based on this, administration of stable iodine before or at the beginning of exposure to radioactive iodine will block the uptake of radioactive iodine by the thyroid because that gland has already been saturated by stable iodine (5). Thus, internal exposure of the thyroid is effectively reduced, **Figure 2**.

Nauman and Wolff (15) estimated a 40-62% radiation dose decrease to the thyroid when KI is administered 1-4 d after the beginning of exposure. Jang et al. (105) calculated a protective effect of KI of 78.9 or 39.1% when KI is administered within 2 h or at 8 h after I^131^ intake, respectively. As a result of iodine blockage of the thyroid gland, also known as ITB, or iodine prophylaxis, the risk of developing thyroid cancer is decreased. To date, it has been established that ITB is effective in reducing the uptake of radioactive iodine by the thyroid gland and thus the subsequent risk of thyroid cancer. In addition, ITB may also reduce the risk of deterministic effects e.g. hypothyroidism caused by tissue damage.

Regardless of chemical form being used, I- iodide ion is the active chemical entity. The iodide ion, for example in the form of KI, acts on the thyroid and prevents radioiodine binding through five different mechanisms, but two are the main ones, namely isotopic dilution by acting as a substrate and diluting circulating radioiodine in the body available for uptake by the thyroid; and saturation of active iodine transport mediated by the NIS located on the surface of thyroid cells (16). Other mechanisms include inhibition of iodide organification, which is potentially responsible for decreased THs synthesis and possible hypothyroidism; this effect is usually short-lived, but the fetus and neonate may be affected; generation of an organic iodine compound that inhibits I^131^ binding; and finally, inhibition of iodine organification secretion by the thyroid. Overall, strategies that can be adopted to reduce the risk of adverse health outcomes in people exposed to an accidental release of radioactive iodine include oral administration of stable iodine and control of food and drinking water. The Chernobyl nuclear reactor accident in 1986 caused a large release of I^131^. Studies in atomic bomb survivors show how thyroid cancers can develop after external exposure to ionizing radiation (106, 107). Higher rates of thyroid cancer have been observed in individuals living in contaminated areas of Belarus, Ukraine, and the western part of the Russian Federation.

Thyroid cancer risk from radioiodine exposure is closely related to the ages of the exposed subjects. The younger the individual exposed at the time of radioiodine contamination, the greater the replicative activity of thyroid cells and the more immature the thyroid regulatory system, the greater the risk of developing thyroid cancer (5, 84). It was also reported that iodine deficiency has been associated with an increased risk of radiation-induced thyroid cancer in populations involved in the Chernobyl accident (13). Specifically, in the case of nutritional iodine deficiency, the thyroid is “iodine-starved” and absorbs more radioactive iodine than it would if there was adequate nutritional iodine intake. Consequently, blocking the thyroid with iodine in countries characterized by iodine deficiency is of paramount significance.

The use of iodine thyroid blockage (ITB) as an urgent protective action in response to radioiodine release was first described in the 1960s and 1970s (108) and addressed in detail in the World Health Organization (WHO) guidelines for iodine prophylaxis following nuclear accidents published in 1989 and revised in 1999 in light of new knowledge regarding the risk of childhood thyroid cancer following the 1986 Chernobyl accident (5). Current guidelines (109) support the general criterion for the use of ITB for an equivalent projected dose to the thyroid of 50 mSv for the first seven days after the onset of exposure. The accident at the Fukushima Daiichi nuclear power plant raised the issue regarding the use of ITB as an urgent protective action. In this case, the 2015 IAEA report on Fukushima (110), highlights the need to standardize the use of the ITB (111). ITB is a defensive measure that is performed only in the urgent phase (hours to a day after the onset of the emergency). Regarding the earliest phase (days to weeks), an effective way to limit radioiodine ingestion (as demonstrated by the Fukushima experience) and the main way to limit thyroid doses, especially to children, is to limit consumption of contaminated food, drinking water, and fresh milk from grass-fed cows. Regulations for ITB implementation must be carefully deliberated in the preparation phase and should include recommendations for: chemical form, packaging, dosage, timing of administration, stocking, distribution and pre-distribution, and identification of appropriate places (e.g., health care facilities, households, schools, workplaces, and daycares). KI is most used to make ITB and thus protect the thyroid from absorbing radioactive iodine. KI is a chemical compound containing iodine which can be administered to protect the thyroid gland from potential damage from radioiodine, which is released during nuclear accidents. KI works by reducing the amount of radioiodine that is concentrated in the thyroid after it is inhaled or ingested through contaminated milk and other foods.

The timing of use is a potential limitation: KI is highly effective in blocking radioiodine uptake if taken shortly before or shortly after exposure. On the positive side, side effects after short-term use have been minimal. However, other chemical forms, such as potassium iodate (KIO3), are equally valid, as long as the dosage is adjusted to contain the same amount of iodine. There is no decisive difference in shelf life between KI and KIO3. Under appropriate storage conditions, packaged tablets retain their iodine content for at least five years. On the other hand, the shelf life is more limited if the stable iodine is in powder or an aqueous solution (5).

UK guidelines state that ITB should be performed if the adsorbed dose to the unblocked thyroid is greater than 50 mGy (112). A stable iodine dosing regime is expressed as either total KI or iodide alone (I-): 130 mg of KI is equivalent to 100 mg of iodide. In normal adults, an oral dose of about 100 mg of iodine reduces thyroid uptake to less than 1% of the normal uptake. Giving a higher, single dose is beneficial because it provides a high level of iodide in the blood, which saturates the iodine pool and is directly related to both blocking efficacy and longer duration of effect.

A single administration of SI is usually sufficient. WHO and FDA have recommended 100 mg of iodine (130 mg of KI) as the standard adult dose for thyroid blockage, which is indicated for pregnant and breastfeeding women too (5, 113). The recommended dose for children 3 to 18 years of age is 65 mg of KI (15). In infants (over 1 month through 3 years of age) the dose should not exceed 32 mg, and from birth to 1 month it should not exceed 16 mg. Nevertheless, adolescents approaching adult size (70 kg and over) should receive the full adult dose (130 mg of KI).

The timing of KI versus radioiodine exposure is critical in determining the efficacy of blockage. Nevertheless, the greatest protection coincides with radioiodine exposure. The recommended timing of SI administration is less than 24 hours prior to, and up to two hours after, the expected onset of exposure. In the case of prolonged (beyond 24 hours) or repeated exposure, unavoidable ingestion of contaminated food and drinking water, and where evacuation is not feasible, repeated administration of SI should be considered daily. The repeated administration is not recommended in neonates, pregnant and breastfeeding women and older adults).

The use of KI after exposure to I^131^ still offers an 80% protective effect, and this has important implications in the event of a nuclear accident.

Stable iodine side effects are rare and include iodine-induced transient hyper- or hypothyroidism, nonthyroidal adverse effects, and allergic reactions (16, 18). The severity of these effects varies according to age, situation, and habitual iodine intake (16). In Poland, Nauman and Wolff (15) conducted a large-scale survey regarding the use of KI in Poland following the Chernobyl accident. Specifically, the doses of KI used were as follows: 15 mg for infants, 50 mg for children aged 5 years or less, and 70 mg for children older than 5 years and for pregnant and lactating women (for adults, iodine prophylaxis was not mandatory) as a single administration. Overall, few adverse events were noted. Notably, no differences in thyroid effects were found between children receiving KI compared with children not receiving KI.

Only Nauman and Wolff (15) and Todd et al. (114), in another study, described extrathyroidal effects evident after intake of large iodine doses for prolonged periods, such as headache, abdominal pain, diarrhea, vomiting, dyspnea, and eczema. More rarely, dermatologic and other effects such as acneiform eruptions and dermatitis, fever, and arthralgias have been highlighted and reported (115). Reactions due to iodide hypersensitivity include minor skin rashes, facial and glottic oedema, eczema, and psoriasis exacerbations. Subjects with preexisting thyroid disorders and hypersensitivity to iodine are considered at risk for such reactions (17, 116). While in Nauman and Wolff’s study (15), 0.2% of the population was estimated to have medically significant adverse effects, conversely, an adverse effect frequency of 5/10000 was estimated by the Food and Drug Administration of the U.S. Department of Health and Human Services. Another point reported in the literature is that KI adverse effects are more likely to occur in iodine-deficient regions where iodine administration may lead to “imbalance” of preexisting functional autonomy and hyperthyroidism, but the available data is poor.

Overall, the risk of developing adverse effects secondary to KI prophylaxis should be related to the characteristics of the population receiving KI, their risk of developing thyroid cancer, and the dose of KI. Benefits and risks should also be considered according to age. The target population is infants, young children, pregnant and breastfeeding women because they are particularly vulnerable to radioactive iodine isotopes (17). However, it is also this segment of the population that most frequently experiences adverse effects from KI, although still at a low rate. Therefore, administration of iodine for prophylactic purposes should be cautious.

In **Table 6**, we reassumed the indications, benefits and risk for a prophylactic use of iodine for thyroid blocking in the event of a nuclear disaster.

**Table 6:** Indications, benefits, and risk of iodine thyroid blockage in case of nuclear disaster.

INDICATIONS	BENEFITS	RISKS
Iodine thyroid blockage is a defensive measure only in the urgent phase	In the event of radiation emergency, the ingestion of stable iodine prevents radioiodine entry and organification limiting radiation exposure to the thyroid	Thyroid cancer risk from radioiodine exposure is related to the dose experienced
The optimal period of administration of SI is less than 24 hours prior to, and up to two hours after, the expected onset of exposure	The stable iodine is effective in preventing the carcinogenesis of thyroid cancer	The susceptibility of the thyroid to develop cancer decreases with age. Infants, young children, pregnant and breastfeeding women are particularly vulnerable
A single administration of SI is usually sufficient	Due to this natural adaption mechanism it’s possible to tolerate large doses of iodine without clinical effects	Prolonged assumption of the iodine can be dangerous and may precipitate in severe thyroidal (iodine-induced transient hyper or hypothyroidism) and non-thyroidal negative effects (allergic reactions, sialadenitis gastrointestinal disturbances, minor rashes).
In the case of prolonged (beyond 24 hours) or repeated exposure, and where evacuation is not feasible, repeated administration of stable should be considered daily		The risk of developing adverse effects secondary to KI prophylaxis is related to the characteristics of the population receiving KI, their risk of developing thyroid cancer, and the KI dose; KI adverse effects occur also more likely in iodine-deficient regions

## Conclusion

Iodine is an essential element in the production of THs. Both deficient and excess SI intakes may precipitate in adverse thyroidal events in children, leading to functional and developmental abnormalities of different body organs, particularly the brain. Therefore, the appropriateness of iodine intake is critical to thyroid health. RI is a common a byproduct of nuclear fission processes, and exposure to ionizing radiation is a known cause responsible for the development of cancer in the human body. As the thyroid gland cannot discern between RI and SI, in the case of a nuclear disaster RI absorption occurs, leading to gland damage and the aforementioned cancer risk.

The use of SI is a means for protection against radioactive iodine and the primary goal is to protect against thyroid cancer. Considering the growth and metabolism of the thyroid gland, age is a significant factor, and children may be a priority target for the administration of stable iodine from the viewpoint of radiation exposure prevention. Precise indications are mandatory to use the iodine for thyroid blocking. The main principle of emergency prophylactic use of SI is a single dose as early as possible, associated with the prompt evacuation from contaminated areas and avoidance of inhalation and ingestion of contaminated materials. Due to this natural adaption mechanism it’s possible to tolerate large doses of iodine without clinical effects. However, a prolonged iodine assumption when not needed can be dangerous and may precipitate in severe thyroidal and, less frequently, non-thyroidal negative effects. In the event of a nuclear disaster, comprehensive measures should be taken both in medical treatment and in support from public health and policy perspectives.

## Author Contributions

VC, CM, and GZ participated in the study design, project management, and supervision. VC, CM, VR, GM, MG, and PB were responsible for the conceptualization and design of forms, data management, writing, and editing. VC, CM, GZ supervised the manuscript. All authors contributed to the article and approved the submitted version.

## Conflict of Interest

The authors declare that the research was conducted in the absence of any commercial or financial relationships that could be construed as a potential conflict of interest.

## Publisher’s Note

All claims expressed in this article are solely those of the authors and do not necessarily represent those of their affiliated organizations, or those of the publisher, the editors and the reviewers. Any product that may be evaluated in this article, or claim that may be made by its manufacturer, is not guaranteed or endorsed by the publisher.
